# Voltage‐Associated Abdominal Pain During Bedside Temporary Transvenous Pacing: A Case Report

**DOI:** 10.1002/ccr3.72466

**Published:** 2026-04-03

**Authors:** Hongling Li, Meng Liu

**Affiliations:** ^1^ Emergency Department Affiliated Hospital of Chifeng University Chifeng Inner Mongolia China; ^2^ Emergency Department Hunan Provincial People's Hospital (The First Affiliated Hospital of Hunan Normal University) Changsha Hunan China

**Keywords:** abdominal pain, complete atrioventricular block, high pacing threshold, pacing complication, phrenic nerve stimulation, temporary transvenous pacing

## Abstract

A high pacing threshold accompanied by voltage‐dependent abdominal pain should raise suspicion of inadvertent coronary sinus lead placement. In such cases, complete withdrawal and re‐advancement of the lead should be considered. As complementary tools, intracavitary ECG and transthoracic echocardiography may be used to confirm correct lead positioning and ensure stable capture.

## 1 Introduction

Temporary transvenous pacing remains a lifesaving intervention for patients with life‐threatening dysrhythmias and hemodynamic instability, particularly in patients presenting with syncope [1]. Although generally safe, malposition of the pacing lead may occur and lead to serious complications. Achieving initial pacing capture is generally not difficult, with reported bedside success rates of 95%–97% [2, 3]. The primary challenge lies in maintaining a stable pacing system and avoiding complications [4]. We present the case of a 54‐year‐old man presenting with a high pacing threshold and voltage‐dependent abdominal pain during temporary transvenous pacing.

## 2 Case History

**FIGURE 1:**
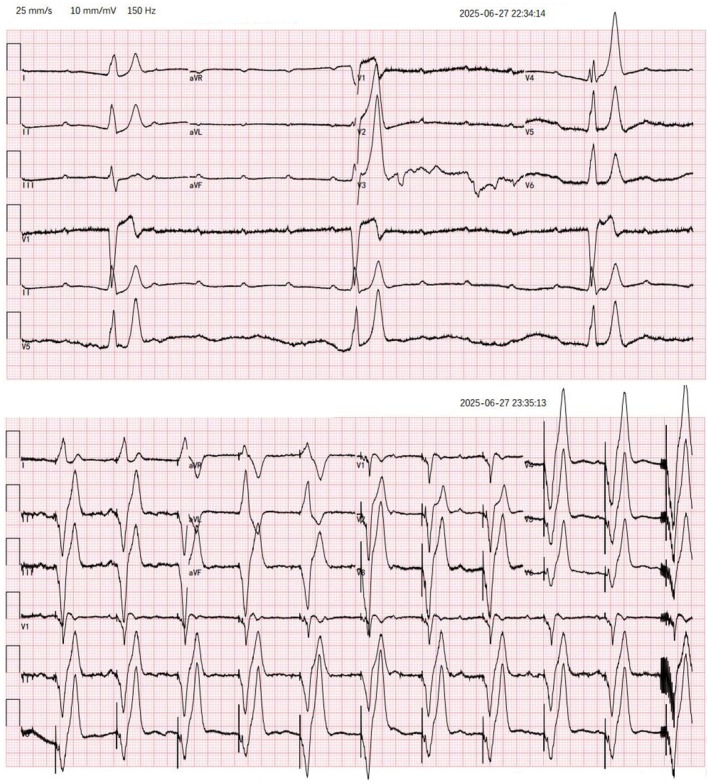
Electrocardiograms before and after temporary transvenous pacing. Baseline ECG showing sinus rhythm with complete atrioventricular block and a ventricular escape rhythm at 17 beats per minute. Post‐pacing ECG demonstrates a typical pattern of right ventricular apical pacing.

A 54‐year‐old male presented to the emergency department after a witnessed syncopal episode during outdoor exercise. Collapse was abrupt, with no preceding chest pain, dizziness, or gastrointestinal symptoms. The patient regained consciousness after approximately 8 min with stimulation from family members and was diaphoretic. He had a 5‐year history of bradycardia of unknown cause and a syncopal episode 2 months earlier, with no hypertension or diabetes. On arrival, vital signs showed a temperature of 36.8°C, ventricular rate of 17 beats per minute, respiratory rate of 20 breaths per minute, unrecordable blood pressure, and oxygen saturation of 96%. He was alert and able to engage in normal conversation. His extremities were cool on palpation, but muscle strength and tone were preserved. Lung auscultation revealed clear breath sounds bilaterally. Electrocardiography revealed sinus rhythm with third‐degree atrioventricular block and a very slow ventricular escape rhythm (Figure 1). Laboratory tests showed troponin I 0.014 ng/mL (normal < 0.023 ng/mL) and normal pro‐BNP.

## 3 Treatments

**FIGURE 2:**
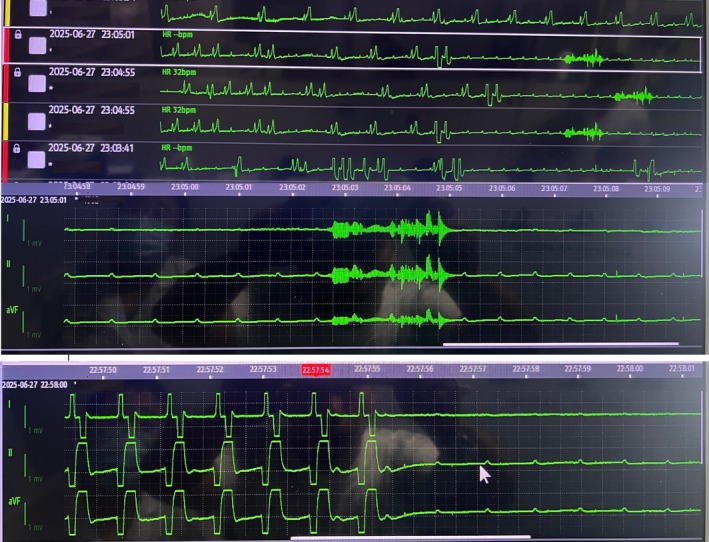
Telemetry review of malignant arrhythmic events. The arrow indicates the initial sudden loss of ventricular capture. Three episodes of prolonged asystole occurred, each showing high‐frequency myoelectric activity at 6.5 s, corresponding to convulsive movements during syncope.

**FIGURE 3:**
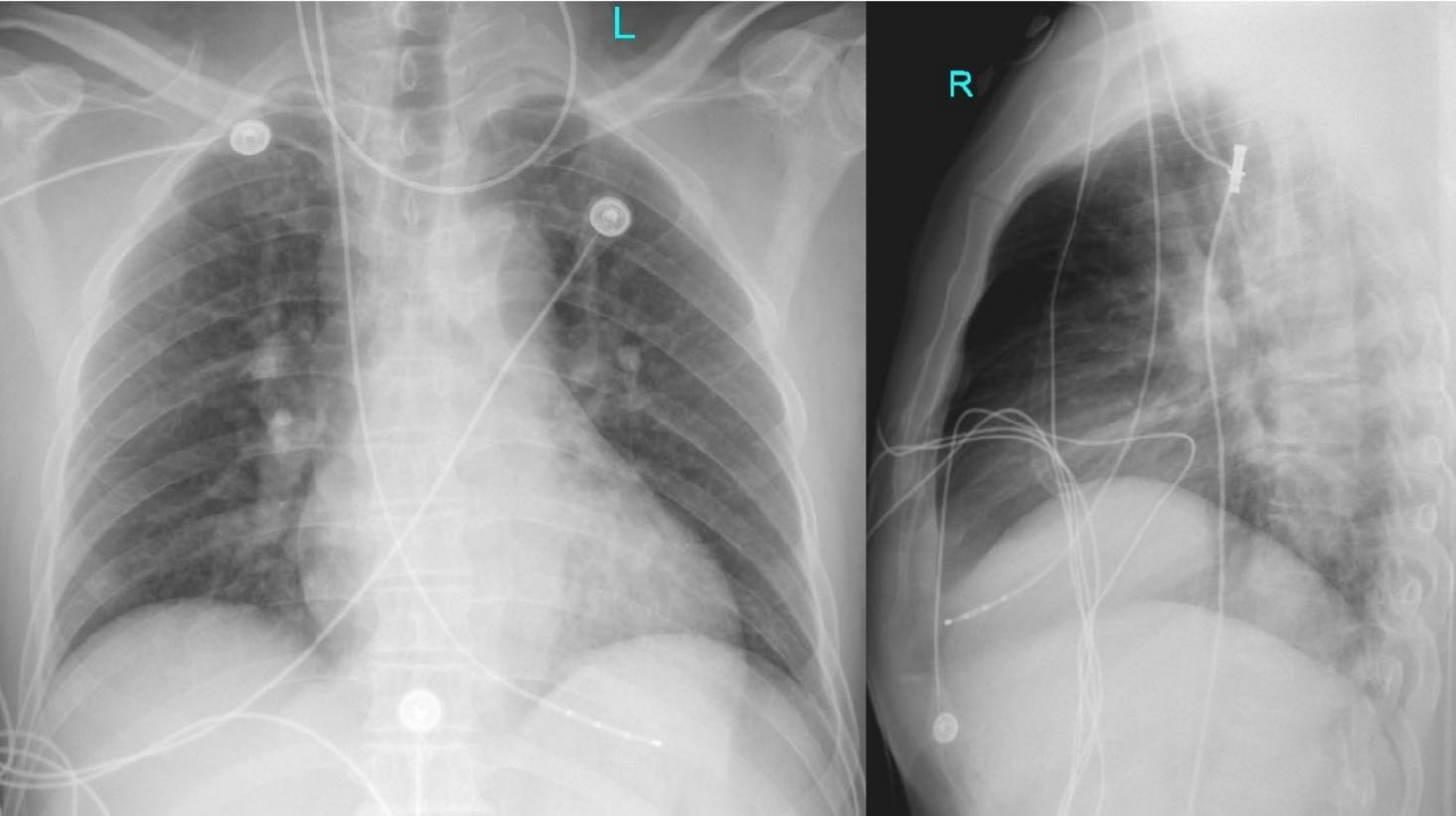
Post‐procedure chest radiograph. Posteroanterior and lateral views confirm the pacing lead positioned at the right ventricular apex.

Given unstable bradycardia, a temporary transvenous pacemaker was placed within 30 min of admission. A 6 French quadripolar pacing catheter with a soft distal 10 cm segment was inserted through the right internal jugular vein via a 7 French introducer sheath. The catheter tip was shaped to 20–30 degrees and oriented toward the patient's left side by a blind technique [5]. The catheter was then advanced with the pacemaker activated until the cardiac monitor showed a broad QRS complex consistent with ventricular pacing, after which it was advanced a further 2–3 cm, for a total insertion length of approximately 30 cm. The pacemaker was set at a rate of 60/min, sensitivity of 2 mV, and output of 5 V. Shortly thereafter, loss of capture occurred (Figure 2). Increasing output to 7 V restored capture, and output was set at 10 V. At this output, the patient developed severe abdominal pain with an ill‐defined component of discomfort; the symptoms subsided when the pacing voltage was reduced, but this resulted in loss of capture. Repeated lead repositioning maneuvers, including rotation and small depth adjustments, failed to achieve stable pacing. During this period, the patient experienced three syncopal episodes with generalized convulsions; telemetry demonstrated ventricular asystole, with high‐frequency myoelectric activity emerging at 6.5 s, corresponding to syncope and convulsive movements (Figure 2). We recognized the possibility that the pacing lead had entered the coronary sinus; the pacing lead was completely withdrawn and then reinserted, resulting in stable ventricular capture (Figure 1) with a pacing threshold of 0.6 V. After stabilization, no focal neurological deficits were noted, and the abdomen was soft without tenderness or guarding. Transthoracic echocardiography in the subcostal four‐chamber view confirmed right ventricular lead position, and chest radiography demonstrated appropriate placement (Figure 3). Subsequent echocardiography and CT examinations demonstrated no evidence of cardiac perforation.

## 4 Conclusions and Results

A permanent pacemaker was implanted on hospital day 3. At one‐month follow‐up, the patient remained clinically stable and asymptomatic.

## 5 Discussion

This case presented an unusually high pacing threshold accompanied by voltage‐dependent abdominal pain during temporary transvenous pacing. Our initial thought was that the pacing lead was positioned within the right ventricle but had poor myocardial contact. In most cases, slight manipulation of the lead—advancing, withdrawing, or rotating—can restore capture; however, this was not achieved here. Because both the cathode and anode of a bipolar pacing lead are confined within the ventricular myocardium, the resulting current field is limited to the intramyocardial region, making stimulation beyond the epicardial surface unlikely. The occurrence of abdominal pain at a pacing output of 10 V therefore suggested possible extracardiac stimulation rather than stable right ventricular endocardial pacing. Since the pacing threshold of the epicardium is much higher than that of the endocardium, successful ventricular capture suggests that the lead was in proximity to the ventricular wall.

Right ventricular perforation was initially considered. However, three findings made this diagnosis unlikely: (1) the distal 10 cm of the pacing catheter used in this case was particularly soft, making mechanical perforation unlikely; (2) the patient's abdominal pain was strictly voltage‐dependent and not associated with pericardial irritation, hemodynamic compromise, or echocardiographic/CT evidence of perforation [6, 7, 8]; and (3) if the lead had penetrated the epicardium, minimal withdrawal should have re‐established stable endocardial capture, which did not occur.

Complete withdrawal and reinsertion resulted in stable pacing at a low threshold, supporting this hypothesis. If the pacing lead enters the coronary sinus and lies near the diaphragm, pacing output may stimulate the phrenic nerve, resulting in abdominal or diaphragmatic discomfort. Such phrenic nerve stimulation is well recognized in permanent pacing [9, 10]. In earlier clinical experience, malposition of a permanent pacing lead into the coronary sinus was not uncommon when the intended target was in the right ventricular apex [11]. For temporary pacing, unintentional entry into the coronary sinus may occur through a similar mechanism. Nevertheless, complications related to this form of malposition appear to be infrequently described in the literature. Two factors may explain this: (1) the procedural endpoint for temporary pacing is simply electrical capture, without routine confirmation of lead position by intracavitary electrocardiography, threshold testing, echocardiography, or multi‐angle radiography as is customary for permanent implants [2, 3]; and (2) when capture is lost, or complications arise, the operator's priority is often rapid repositioning rather than immediate imaging confirmation. A limitation of this case is the lack of imaging evidence confirming coronary sinus entry; therefore, the proposed mechanism remains presumptive. Bedside echocardiography may help assess lead position, and in some cases, coronary sinus entry can be suggested by an abnormal lead course in apical or subcostal four‐chamber views [4].

This case highlights a key teaching point: a high pacing threshold combined with voltage‐dependent abdominal symptoms should raise suspicion of pacing lead malposition, including possible coronary sinus entry. If suspected, the lead should be withdrawn sufficiently to exit the coronary sinus before re‐advancement into the right ventricle. Whenever feasible, correct lead positioning with confirmed right ventricular endocardial contact—preferably verified by intracavitary ECG before pacing initiation—helps ensure stable pacing capture and prevents pacing instability and subsequent life‐threatening asystole, without adding procedural complexity or time [4, 11]. Bedside transthoracic echocardiography may further assist in visualizing lead advancement and confirming appropriate right ventricular positioning [4].

## Author Contributions

**Hongling Li:** data curation, investigation, visualization, writing – original draft. **Meng Liu:** conceptualization, formal analysis, methodology, supervision, writing – review and editing.

## Funding

The authors have nothing to report.

## Ethics Statement

Patient information has been anonymized, and written informed consent was obtained for publication.

## Conflicts of Interest

The authors declare no conflicts of interest.

## Data Availability

The data supporting the findings of this study are available within the article. No additional data are available.

## References

[1] F. M. Kusumoto , M. H. Schoenfeld , C. Barrett , et al., “ACC/AHA/HRS Guideline on the Evaluation and Management of Patients With Bradycardia and Cardiac Conduction Delay: A Report of the American College of Cardiology/American Heart Association Task Force on Clinical Practice Guidelines and the Heart Rhythm Society,” Circulation 140, no. 8 (2018): e382–e482, 10.1161/CIR.0000000000000628. 30586772

[2] R. H. Birkhahn , T. J. Gaeta , J. Tloczkowski , et al., “Emergency Medicine‐Trained Physicians Are Proficient in the Insertion of Transvenous Pacemakers,” Annals of Emergency Medicine 43, no. 4 (2004): 469–474, 10.1016/j.annemergmed.2003.09.019. 15039689

[3] V. Gangathimmaiah , “Emergency Transvenous Cardiac Pacing,” Emergency Medicine Australasia 29, no. 2 (2017): 229–232, 10.1111/1742-6723.12757. 28296230

[4] P. Blanco , “Temporary Transvenous Pacing Guided by the Combined Use of Ultrasound and Intracavitary Electrocardiography: A Feasible and Safe Technique,” Ultrasound Journal 11, no. 1 (2019): 8, 10.1186/s13089-019-0122-y. 31359249 PMC6638614

[5] M. Liu and X. Han , “Bedside Temporary Transvenous Cardiac Pacemaker Placement,” American Journal of Emergency Medicine 38, no. 4 (2020): 819–822, 10.1016/j.ajem.2019.12.013. 31864866

[6] K. Omote , T. Aikawa , Y. Ishidoya , D. Sunaga , and N. Funayama , “Temporary Transvenous Pacemaker Lead‐Induced Cardiac Perforation Incidentally Detected by Right Ventriculography Prior to Leadless Pacemaker Implantation,” Cureus 17, no. 5 (2025): e84136, 10.7759/cureus.84136. 40530213 PMC12170247

[7] D. W. Barron , N. G. Rasmussen , M. H. Auerbach , D. Rappaport , and W. A. Martini , “Pacemaker Lead Perforation Presenting as Persistent Abdominal Pain: A Case Report,” Cureus 17, no. 5 (2025): e84116, 10.7759/cureus.84116. 40519404 PMC12166111

[8] C. Menexi and M. ElRefai , “Pacemaker‐Lead Dislodgement and Cardiac Perforation,” New England Journal of Medicine 390, no. 19 (2024): 1802, 10.1056/NEJMicm2312569. 38749035

[9] J. Julia , M. Lopez‐Gil , A. Fontenla , et al., “Super‐Response to Cardiac Resynchronization Therapy May Predict Late Phrenic Nerve Stimulation,” Europace 20, no. 9 (2018): 1498–1505, 10.1093/europace/eux311. 29182757

[10] G. Moubarak , A. Bouzeman , J. Ollitrault , F. Anselme , and S. Cazeau , “Phrenic Nerve Stimulation in Cardiac Resynchronization Therapy,” Journal of Interventional Cardiac Electrophysiology 41, no. 1 (2014): 15–21, 10.1007/s10840-014-9917-8. 24934757

[11] S. J. Gulotta , “Transvenous Cardiac Pacing. Technics for Optimal Electrode Positioning and Prevention of Coronary Sinus Placement,” Circulation 42, no. 4 (1970): 701–718, 10.1161/01.cir.42.4.701. 11993310

